# Milk amyloid A as a biomarker for diagnosis of subclinical mastitis in cattle

**DOI:** 10.14202/vetworld.2018.34-41

**Published:** 2018-01-19

**Authors:** Hany Ahmed Hussein, Khaled Abd El-Hamid Abd El-Razik, Alaa Mohamed Gomaa, Mohamed Karam Elbayoumy, Khaled A. Abdelrahman, H. I. Hosein

**Affiliations:** 1 Department of Animal Reproduction and AI, Veterinary Division, National Research Centre, Dokki, Giza, Egypt; 2 Department of Mastitis and Neonatal Diseases, Animal Reproduction Research Institute, Agriculture Research Center, Giza, Egypt; 3 Department of Parasitology and Animal Diseases, National Research Centre, Dokki, Giza, Egypt; 4 Department of Veterinary Medicine, Faculty of Veterinary Medicine, Beni-Suef University, Beni Suef, Egypt

**Keywords:** biomarkers, milk amyloid A, somatic cell count, subclinical mastitis

## Abstract

**Background and Aim:**

Mastitis is one of the most vital noteworthy monetary risks to dairy ranchers and affects reproductive performance in dairy cattle. However, subclinical mastitis (SCM) negatively affects milk quality and quantity and associated with economic losses as clinical mastitis. It is recognizable only by additional testing. Somatic cell count (SCC) is currently used worldwide for the screening of intramammary infection (IMI) infections. However, somatic cells (SC) are affected by numerous factors and not always correlate with infection of the udder. Therefore, the aim of the present study was to evaluate the milk amyloid A (MAA) in the milk of normal and SCM cows and compare the sensitivity of both MAA secretion and SCC in response to mammary gland bacterial infection.

**Materials and Methods:**

A total of 272 quarter milk samples collected from 68 Friesian cows after clinical examination for detection of clinical mastitis were employed in this study. All quarter milk samples (272) were subjected to bacteriological examination, while SCs were assessed in samples (220). Following SCC estimation and bacteriological examination, the apparently normal quarter milk samples were categorized into 7 groups and MAA concentration was estimated in normal and subclinical mastitic milk samples.

**Results:**

Prevalence of clinical mastitis was 19.12 % (52 quarters), while 80.88 % (220 quarters) were clinically healthy with normal milk secretion. Of those 220 clinically healthy quarter milk samples, 72 (32.73%) showed SCM as detected by SCC (SCC ≥500,000 cells/ml). The most prevalent bacteria detected in this study were streptococci (48.53%), Staphylococcus aureus (29.41%), Escherichia coli (36.76%), and coagulase-negative staphylococci (11.76%). Results of MAA estimation revealed a strong correlation between MAA secretion level and SCC in agreement with the bacteriological examination. Interestingly, there was a prompt increase in MAA concentration in Group III (G III) (group of milk samples had SCC ≤200,000 cells/ml and bacteriologically positive) than Group I (G I) (group of milk samples with SCC ≤500,000 cells/ml and bacteriologically negative), as MAA concentration in G III was about 4 times its concentration in G I.

**Conclusion:**

Our study provides a strong evidence for the significance of MAA measurement in milk during SCM, and MAA is more sensitive to IMI than SCC. This can be attributed to rapid and sensitive marker of inflammation. The advantage of MAA over other diagnostic markers of SCM is attributed the minute or even undetectable level of MAA in the milk of healthy animals, it is not influenced by factors other than mastitis, and could be estimated in preserved samples. Therefore, we recommend that estimation of MAA concentration in milk is a more useful diagnostic tool than SCC to detect SCM and to monitor the udder health in dairy cattle.

## Introduction

Mastitis is an inflammation of mammary gland, caused by several types of bacteria and their toxins [1]. It is the most prevalent disease causing great economic losses in dairy cattle because of a decrease in milk yield, altered compositional quality, discarded milk, cost of treatment and veterinary care, and increased involuntary culling rates [2,3]. Mastitis causes physical, chemical, and microbial changes in the secreted milk with pathological alterations in the glandular tissue of the udder [4]. Furthermore, bacterial contamination in mastitic milk renders milk unfit for human consumption [5,6].

Beyond its negative impact on milk yield and milk components, mastitis has a detrimental effect on reproductive performance in dairy cows [7-11].

In dairy cattle and buffaloes, the prevalence of mastitis may exceed 50% [4] with 15-40 more times the incidence of subclinical mastitis (SCM) than clinical mastitis [12].

SCM does not cause any visible changes in milk or udder appearance but affects milk quality and quantity causing a reduction in milk yield up to two-third losses of the total milk production [13,14], altered milk composition, and the presence of inflammatory components and bacteria in milk. Furthermore, cows suffering from SCM produce visibly normal milk and are a source of infection for other animals, resulting in the spread of infection among the herd [15,16]. SCM is difficult to be detected by visual inspection and palpation of the udder due to the absence of visible changes in the udder or milk, which makes SCM more challenging.

Hence, detection and prompt therapeutic strategies of SCM would be helpful not only for minimizing the possibility of the spread of infection but also to maintain good reproductive efficiency.

Several methods exist of diagnosis of mastitis, and bacteriologic examination of milk samples is considered the standard method [17]; however, it is expensive and time-consuming. Otherwise, other diagnostic methods, as somatic cell count (SCC), are currently used for screening of intramammary infections (IMI) [18].

SCC is used as an indicator for milk quality [19]. Milk from healthy animals contains low levels of somatic cells (SC). Increased number of these cells indicates abnormal milk secretion with inferior quality that is caused by an IMI (mastitis), and the major factor elevates SCC [20].

SCC has been commonly used worldwide for decades as the gold standard indicator for SCM and to assess the effectiveness of mastitis control programs in dairy herds [21]. However, SCs are affected by numerous factors, of which, age, lactation period, parity, season, stress, management, and day-to-day variation [22]. Therefore, SCC does not always correlate with infection of the udder[23], the matter which clarifies the need for other supplementary or substituting tools for SCC.

The interest in research for biomarkers to be used for diagnosis of mastitis in cattle stems from the need to better characterization of the mechanisms of the disease [24,25].

A clear understanding of mastitis pathogenesis is necessary for the development of adequate tools utilized for mastitis diagnosis [26]. Pathogenesis of mastitis involves an inflammatory reaction resulting from response to many factors including microorganisms overcoming the physical barriers of the teat canal. Once get inside the teat cistern, the bacteria start multiplication [27], and the innate immune system is induced through contact of these invading bacteria with SC in the milk and the lining epithelial cells [28-31], followed by upregulation of cytokine production [32]. These cytokines attract neutrophils to the site of inflammation [33], induce expression of vascular endothelial adhesion molecule, and promote neutrophil transendothelial migration to the site of infection [34]. Some of these cytokines induce the acute-phase response, which is characterized by fever, leukocyte mobilization, and increased production of acute-phase proteins (APP) [27].

Nowadays, APPs have become important diagnostic biomarkers in human medicine, as well as, in veterinary diagnostics [35]. Interest in APPs as potential biomarkers in veterinary medicine involves the evaluation of their concentration and modifications, as well as, their interaction as a part of the host response [36-39].

Serum amyloid A (SAA) and haptoglobin (Hp) are the major APPs in ruminants [40,41]. The prompt and intense increase of SAA concentration in plasma and/or other body fluids (1000 times) shortly (24 h) after tissue injury [42,43] makes this protein potentially useful as non-specific inflammation marker, monitoring health status, and evaluating responses to primary and adjunctive therapy in veterinary practice [44]. SAA was reported to have multiple pro-inflammatory and anti-inflammatory activities [45]. Levels of this protein in milk have been proposed as a sensitive indicator of mastitis infection in the dairy cows [46,47].

In addition to the above-mentioned facts, the currently available diagnostics used for detection of mastitis, especially in the early stage of disease, might be confused with other physiological disorders. Therefore, the aim of the present study was directed to evaluate the milk amyloid A (MAA) secretion in the milk of normal and SCM cows with different udder health and infection status to be used as a biomarker either as an alternative or supplementary for SCC tests for SCM detection.

## Materials and Methods

### Ethical approval

All samples were collected as per standard sample collection procedure without giving any stress or harm to the animals. The present work was approved by the Ethical Committee for medical research at the National Research Center and Animal Care guidelines of the General Organization for Veterinary Services, Egypt.

### Animals

A total of 68 Friesian cows, in the mid-lactation period, located in Damietta Governorate, Egypt, were employed in this study. These animals were subjected to clinical examination for detection of any clinical abnormalities with special attention to the udder by visual inspection and palpation for detection of clinical mastitis according to Kelly [48]. Clinical mastitis was considered in case of pain on milking, swelling of the udder, a decrease in milk production, and changes in milk (yellowish color or presence of flakes).

### Samples

A total of 272 quarter milk samples collected from 68 Friesian cows were employed in this study where 15 ml of milk were collected in the sterile tube under strict hygienic measures from each quarter after disinfection of the teat with 70% alcohol. The first 3 squirts from each quarter were discarded. Milk samples were kept on the ice and transferred immediately to the laboratory for assessment of SCC and bacteriological examination within 24 h. Samples were stored at −18°C until the MAA assay was performed.

### SCC

Milk SCC was assessed in 220 apparently normal quarter milk samples by The NucleoCounter^®^ SCC instrument that is based on ChemoMetec’s proven technology of fluorescence image cytometry. This method uses the single-use SCC-Cassette™ sampling and measuring device, the NucleoCounter^®^ SCC-100™ system. The measurement range of the NucleoCounter^®^ SCC-100™ is between 1×10^4^ cells/ml and 2×10^6^ cells/ml, with an optimal measurement range of between 1×10^4^ cells/ml and 1×10^6^ cells/ml.

### Bacteriological examination

Bacteriological examination of milk samples was performed according to Malinowski and Kołosowska [49]. Briefly, 10 μl of milk were cultivated on Blood Agar Base (bioMlrieux Poland), MacConkey Agar (BTL, Poland), Mueller-Hinton Agar (BTL, Poland), and Edwards Medium (Oxoid Ltd., England). Plates were incubated at 37°C and read at 24 and 48 h later. Colonies were identified by their colony morphology and Gram-staining. Detailed identification of isolated bacteria was performed using API tests (bioMerieux Poland).

### Categorization of milk samples according to udder health and infection status

Following SCC estimation and bacteriological examination, samples were categorized according to SCC concentration and microbial isolation into 7 groups as follows:

- Group I (G I) represents bacteriologically negative samples with SCC ≤500,000 cells/ml milk.
- Group II (G II) represents bacteriologically negative samples with SCC ≥500,000 cells/ml milk.
- Group III (G III) represents bacteriologically positive samples with SCC ≤200,000 cells/ml milk.
- Group IV (G IV) represents bacteriologically positive samples with SCC ≥200,000 ≤500,000 cells/ml milk.
- Group V (G V) represents bacteriologically positive samples with SCC ≥500,000 ≤1,000,000 cells/ml milk.
- Group VI (G VI) represents bacteriologically positive samples with SCC ≥1,000,000 ≤2,000,000 cells/ml milk.
- Group VII (G VII) represents bacteriologically positive samples with SCC ≥2,000,000 cells/ml milk.

### Estimation of MAA concentration in milk from the different groups

**Figure-1:**
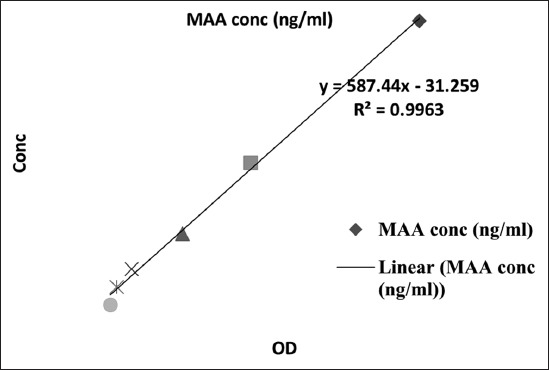
Milk amyloid A standard curve.

The concentration of MAA was determined in five milk samples selected from each of the above-mentioned seven groups using a commercially available enzyme-linked immunosorbent assay (ELISA) kit (Tridelta Mast ID range MMA assay, Tridelta Development Ltd., Kildare, Ireland, Cat. No.: TP-807), as described by McDonald *et al*. [50]. The assay was carried out according to the manufacturer’s recommended protocol. Briefly, standards or samples plus biotinylated monoclonal SAA antibody were incubated in microtiter plate wells pre-coated with capture monoclonal SAA antibody. In one step, MAA in the standard or sample was captured and labeled in a sandwich format. After washing to remove all of the unbound material, wells were incubated with streptavidin-horseradish peroxidase before the addition of enzyme substrate (3,3’,5,5’-tetramethylbenzidine). The reaction was stopped with the addition of kit stop solution. Optical density in the wells was measured at 450 nm using an automated plate reader (Versamax; Molecular Devices, CA, USA). A standard curve was constructed by plotting MAA concentration versus optical density for determining the unknown MAA concentrations of samples (Figure-1). Samples were initially diluted to 1:50 for assay then loaded in duplicate.

### Statistical analysis

Data were presented as means±standard error of the mean. Statistical analyses of the data were performed using SPSS^®^ software [51]. Analysis of variance by simple one-way ANOVA was used to compare the concentration of MAA in milk samples in different udder health and infection status. Duncan’s multiple range test was used to differentiate between significant means at p<0.05.

## Results

Clinical examination of the udder of 68 Friesian cows (272 quarters) revealed the presence of symptoms suggestive for clinical mastitis in 52 quarters (19.12%), while 220 quarters (80.88%) were clinically healthy with normal milk secretion. Pain on milking, swelling of the udder, a decrease in milk production, and changes in milk (yellowish color or presence of flakes) were the most clinically observed symptoms.

The obtained results revealed 68 (25%) bacteriologically negative samples and 204 (75%) bacteriologically positive samples including 96 (35.3%) samples infected with the single pathogen and 108 (39.7%) samples showed mixed infection (Table-1). A total number of 344 bacterial isolates recovered from these 272 quarter milk samples of 68 lactating cows (Table-2). Among the 52 clinically mastitic quarters, 40 (76.92) were bacteriologically positive. The identified pathogens in this study were streptococci (48.53%), *Staphylococcus aureus* (29.41%), *Escherichia coli* (36.76%), and coagulase-negative staphylococci (CNS) (11.76%) (Table-2).

**Table 1:** Results of bacteriological examination of 272 quarter milk samples of 68 lactating cows.

Bacteriological status	Number of samples (%)
Negative samples	68 (25)
Single pathogen	96 (35.3)
Mixed infection	108 (39.7)
Total	272 (100)

204 quarter milk samples were bacteriologically positive

**Table 2:** The identified pathogens with their prevalence rate in quarter milk samples.

Identified bacteria	Number of samples (%)
*S. aureus*	80 (29.41)
CNS	32 (11.76)
*E. coli*	100 (36.76)
Streptococci	132 (48.53)
Total	344

*S. aureus*=*Staphylococcus aureus*, *E. coli*=*Escherichia coli*, CNS=Coagulasenegative staphylococci

SCC revealed that, of the examined 220 apparently normal quarter milk samples, 148 (67.27%) milk samples had SCC ≤500,000 cells/ml and 72 (32.73%) milk samples had SCC ≥500,000 cells/ml (Table-3). Of those having SCC ≤500,000 cell/ml, 100 (67.57%) samples were positive for bacteriological examination. On the other hand, 8 (11.11%) milk samples of those having SCC ≥500,000 cells/ml were bacteriologically negative.

**Table 3:** Results of SCC estimation in 220 apparently healthy quarter milk samples.

SCC	N (%)	Negative for bacteriology	Positive for bacteriology
SCC≤500,000	148 (67.27)	48 (32.43)	100 (67.57)
SCC≥500,000	72 (32.73)	8 (11.11)	64 (88.89)

164 quarter milk samples were bacteriologically positive. SCC=Somatic cell count

**Figure-2:**
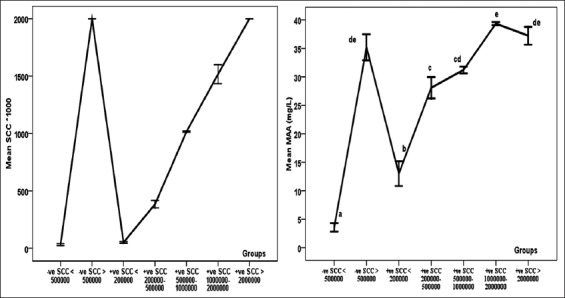
Mean somatic cell count (left) and milk amyloid A (mg/L) (right) in the in seven different mammary health status with error bars. Means with different superscripts (a, b, c, d, and e) are significantly different at p<0.05.

Result of MAA estimation in the 7 investigated groups revealed that the mean MAA concentration was 3.58 mg/ml, 35.2 mg/ml, 13.01 mg/ml, 28.07 mg/ml, 31.23 mg/ml, 39.35 mg/ml, and 37.22 mg/ml in G I, G II, G III, G IV, G V, G VI, and G VII, respectively (Table-4). Results of estimation of MAA in quarter milk samples showed a strong correlation between SCC results and MAA (Figure-2).

**Table 4:** MAA mean concentration (mg/l)±SEM in the 7 different mammary health status.

Sample category	MAA mean concentration/group (mg/l)±SEM
Group I (bacteriologically negative with SCC<500,000 cell/ml milk)	3.58±0.74060
Group II (bacteriologically negative with SCC>500,000 cell/ml milk)	35.2±2.29217
Group III (bacteriologically positive with SCC<200,000 cell/ml milk)	13.01±2.16036
Group IV (positive with SCC 200,000-500,000 cell/ml milk)	28.07±1.87502
Group V (bacteriologically positive with SCC 500,000-1,000,000 cell/ml milk)	31.23±0.58905
Group VI (bacteriologically positive with SCC 1,000,000-2,000,000 cell/ml milk)	39.35±0.26271
Group VII (bacteriologically positive with SCC>2,000,000 cell/ml milk)	37.22±1.57531

SCC=Somatic cell count, MAA=Milk amyloid A, SEM=Standard error of mean

## Discussion

Mastitis continues to gather attention in veterinary research due to its negative impact on milk quantity and components, besides it affects reproductive performance particularly in cattle that represent the largest source of milk production in the world.

In this study, clinical examination of the udder of 68 Friesian cows (272 quarters) proved clinical mastitis in 19.12% of quarters according to Fogsgaard *et al*. [52], while the prevalence of SCM was 32.73% through SCC estimation in the apparently normal milk secretion.

Previous studies concluded that prevalence of bovine mastitis ranged from 29.34% to 78.54% in cows [53], with several times more incidence of subclinical affections than clinical ones [12]. SCM was found to be ranged from 21% to 53% with an average of 36.7% [54]. In 2015, a study carried out in Egypt revealed that prevalence of clinical mastitis was 8.8 % in examined cattle and buffaloes, while the prevalence of SCM was 71.6% in cattle and 43.5 % in buffaloes [55].

The observed decreased milk yield during IMI was explained by Petersson-Wolfe *et al*. [56] that an influx of neutrophils will pass between the milk-producing cells of the mammary gland and into the lumen of the alveoli resulting in damage of milk-secreting cells.

IMI has been proven to increase SCC, change milk quality, decrease milk production, and damage udder tissue [3,4], so SCs are indicators, used for monitoring SCM occurrence in herds or individual cows [1,57]. Secreted SCs in the milk of a healthy cow belongs to the macrophages, neutrophils, mononuclear, and epithelial cells. Neutrophils represent 1-11% of the secreted SCC in the milk of a healthy quarter and increase up to 90% in a quarter with IMI [58].

SCC limit in a healthy quarter is variable among previous studies. For instance, it was stated to be 500,000 cells/ml [57]. Others reported that an increase of SCC above 250,000-300,000 cells/ml was considered abnormal and considered an indication of bacterial infection [59]. Bytyqi *et al*. [54] recorded that milk from a healthy animal had SCC lower than 1×10^5^ cells/ml, and SCC was more 1×10^6^ cells/ml in case of IMI. Moreover, Moroni [55] in Italy found that milk from all quarters with SCC>200,000 cells/ml had IMI, whereas 98% of quarters with SCC below this threshold were uninfected. Despite these obtained results indicate that SCC is a useful predictor of IMI, the affection of SC numerous factors as age, lactation period, parity, season, stress, management, and day-to-day variation [22] is the main obstacle.

The identified pathogens were streptococci, *S. aureus*, *E. coli*, and CNS, with the prevalence of 48.53%, 29.41%, 36.76%, and 11.76%, respectively (Table-2).

In similar studies concerned with identification of the prevalence of SCM causing pathogens, *S. aureus* was identified to be ranged from 0% to 35.7%, streptococci ranged from 7% to 55.5%, *E.coli* ranged from 0% to 10.5%, and CNS ranged from 11% to 60% in examined herds [54]. Furthermore, *S. aureus* was identified in 38.3% of SCM cases in Egypt [55]. In Germany, CNS was isolated from 9% of the quarter milk samples from 80 dairy herds [60]. Kalmus *et al*. [61] recorded the prevalence of streptococci as 30.3%, *E. coli* (15.9%), *S. aureus* (20%), and CNS (15.4%).

The incompatibility between the low SCC and positive bacteriological examination in 100 milk samples (Table-2) might be ascribed to the identification of IMI in the very early stage of infection and recruitment of neutrophils to the site of infection has not been fully achieved. On the other hand, those negative for bacteriology and had high SCC, 8 milk samples (Table-2), as well as the bacteriologically negative 12 samples of the clinical mastitis cases, could be explained by infection with other pathogens as mycotic or mycoplasma infections that failed to be detected with the utilized specific media.

The present study aimed to evaluate the diagnostic value of MAA, as an APP, for detection of IMI in the early stage of infection and compare the sensitivity of both MAA secretion and SCC in response to mammary gland bacterial infection.

The result of MAA estimation in those 7 groups of different udder health status revealed a strong positive correlation and direct proportion between MAA concentration and SCC in accordance with bacteriological examination (Table-4 and Figure-2).

Among the interesting finding obtained in this study, there was a significant prominent change in MAA concentration G III (group of milk samples had SCC ≤200,000 cells/ml and bacteriologically positive) than G I (group of milk samples with SCC ≤500,000 cells/ml and bacteriologically negative), as MAA concentration in G III was about 4 times its concentration in G I (Table-4).

In general, the concentration of Hp or SSA increases in circulation as is a non-specific marker for inflammation anywhere in the animal. However, to provide relevant information about udder health, it must be detected in milk [44]. MAA has been described in the milk of different species, including the cow, horse, and sheep. This form of the protein produced by mammary epithelial cells is abundant in colostrum. However, in milk from healthy animals, the levels are low [62].

Detailed functions of the APPs are not fully understood. APPs are suggested to be engaged in opsonization and trapping of microorganisms, binding of cellular remnants, complement activation, neutralization of enzymes, and elimination of free radicals and hemoglobin [63]. The local rapid and significant increase in MAA concentration as an initial response to inflammation was confirmed in the previous hypothesis that changes in the centralizations of APPs are an early and exceedingly response occurs in the body in case of damage as a mechanism to keep homeostasis and hinder microbial development before creation of the acquired immune response [64]. Besides, MAA has been suggested to have an important protective role in the gastrointestinal tract of the neonate and/or the healthy maintenance of the mammary gland [43,65]. SAA and Hp were recorded to have antibacterial effects [66,67]. This might explain the prominent increase in the concentration of MAA in G III in response to bacterial infection than in G I.

The obtained results coincide with those reported by Safi *et al*. [68] and Pyörälä *et al*. [69] who found that MAA is a reliable biomarker for SCM. Besides, MAA is more sensitive to IMI than SCC. Finally, the MAA-ELISA is considered as a valuable addition to the existing practical applicable tools used for detection of SCM.

## Conclusion

This study provides a strong evidence for the significance of MAA measurement in milk during SCM. This can be a rapid and sensitive marker of inflammation. The advantage of MAA over other diagnostic markers of SCM is attributed to the minute or even undetectable level of MAA in the milk of healthy animals, it is not influenced by factors other than mastitis, and could be estimated in preserved samples. Therefore, we recommend that estimation of MAA concentration in milk is a more useful diagnostic tool than SCC to detect SCM and to monitor the udder health in dairy cattle.

### Authors’ Contributions

HAH, KAA1, and KAA3 designed and planned for this study. HAH, KAA3, and MKE performed the field work and collected the samples. AMG and MKE were responsible for bacteriological examination and SCC. HAH, MKE, and KAA1 carried out ELISA. KAA1 and HIH interpreted the results and reviewed the article. HAH and HIH drafted the manuscript. All authors read and approved the final manuscript.
